# Homocysteine and mitochondrial quality control in diabetic retinopathy

**DOI:** 10.1186/s40662-023-00362-1

**Published:** 2024-01-16

**Authors:** Pooja Malaviya, Renu A. Kowluru

**Affiliations:** Department of Ophthalmology, Visual Sciences and Anatomical Sciences, Wayne State University, 4717 St. Antoine, Detroit, MI 48201 USA

**Keywords:** Diabetic retinopathy, Homocysteine, Hydrogen sulfide, Mitochondria, Mitophagy, Retina

## Abstract

**Background:**

Diabetic retinopathy is a progressive disease, and one of the key metabolic abnormalities in the pathogenesis of diabetic retinopathy, mitochondrial damage, is also influenced by the duration of hyperglycemia. Mitochondrial quality control involves a coordination of mitochondrial dynamics, biogenesis and removal of the damaged mitochondria. In diabetes, these processes are impaired, and the damaged mitochondria continue to produce free radicals. Diabetic patients also have high homocysteine and reduced levels of hydrogen sulfide, and hyperhomocysteinemia is shown to exacerbate diabetes-induced mitochondrial damage and worsen their dynamics. This study aims to investigate the temporal relationship between hyperhomocysteinemia and retinal mitochondrial quality control in diabetic retinopathy.

**Methods:**

Human retinal endothelial cells incubated in 20 mM d-glucose for 24 to 96 h, in the absence or presence of 100 µM homocysteine, with/without a hydrogen sulfide donor GYY4137, were analyzed for mitochondrial ROS (MitoSox fluorescence), DNA damage (transcripts of mtDNA-encoded *ND6* and *CytB*), copy numbers, oxygen consumption rate (Seahorse XF analyzer) and mitophagy (mitophagosomes immunofluorescence labeling and flow cytometry). Results were confirmed in the retina from mice genetically manipulated for hyperhomocysteinemia (cystathionine β-synthase deficient mice, *Cbs*^+/−^), streptozotocin-induced diabetic for 8 to 24 weeks. At 24 weeks of diabetes, vascular health was evaluated by counting acellular capillaries in the trypsin digested retinal vasculature and by fluorescein angiography.

**Results:**

Homocysteine, in high glucose medium, exacerbated mitochondrial ROS production, mtDNA damage and impaired mitochondrial respiration within 24 h, and slowed down/worsened mitochondrial biogenesis and mitophagy, as compared to 48 to 96 h in high glucose alone. GYY4137 supplementation ameliorated homocysteine + high glucose-induced mitochondrial damage and impairment in biogenesis and mitophagy. Similar results were obtained from *Cbs*^+/−^ mice-mitochondrial ROS, mtDNA damage and decline in biogenesis and mitophagy were observed within eight weeks of diabetes vs. 16 to 24 weeks of diabetes in *Cbs*^+/+^ mice, and at 24 weeks of diabetes, *Cbs*^+/−^ mice had significantly higher acellular capillaries and vascular leakage.

**Conclusions:**

Hyperhomocysteinemia, in a hyperglycemic environment, overwhelms the mitochondria, accelerating and exacerbating their dysfunction, and also delays/worsens their removal, augmenting the development of diabetic retinopathy. Thus, our results strengthen the importance of maintaining homocysteine-hydrogen sulfide balance during the early stages of diabetes for a patient to prevent/retard vision loss.

## Background

Diabetes is now considered an epidemic of the 21st century, and among its many chronic complications, damage to the retina leads to vision loss; in fact, diabetic retinopathy is the leading cause of vision loss in working age adults. Pathogenesis of diabetic retinopathy is complex and involves many biochemical, molecular, functional and histopathological changes in the retina and its vasculature, induced by hyperglycemia [1–3]. Experimental models have documented the importance of mitochondrial homeostasis in its development, and have shown that retinal mitochondrial structure, function, DNA and biogenesis are damaged in diabetes, resulting in a vicious cycle of free radicals [2, 4]. Diabetic retinopathy is a progressive disease, and although increase in retinal cytosolic reactive oxygen species (ROS) production can be seen within two weeks of induction of diabetes in rodents, increase in mitochondrial damage is not observed till the duration of diabetes is extended beyond four months [5, 6]. Mitochondrial damage is intimately associated with accelerated apoptosis of retinal capillary cells, which precedes the development of retinal vascular pathology including degeneration of capillaries (acellular capillaries), characteristic of diabetic retinopathy [2, 4].

To ensure mitochondrial quality control while maintaining the necessary number of healthy mitochondria, the coordination of mitochondrial dynamics, biogenesis and removal of damaged mitochondria is crucial [7]. Damaged mitochondria result in the accumulation of more free radicals, creating a toxic environment for cell survival, which makes their removal essential for cell survival [8]. Mitochondria-selective autophagy, ‘mitophagy’ facilitates removal of the damaged mitochondria [9–11], and in diabetic retinopathy, mitochondrial biogenesis, dynamics and mitophagy are impaired, resulting in the accumulation of damaged mitochondria [12, 13].

Diabetic patients usually have high levels of circulating homocysteine, a non-protein forming sulfur-containing amino acid which is synthesized during the methionine metabolism, and high homocysteine is implicated in many diabetic complications including cardiac dysfunction and retinopathy [14–16]. Homocysteine is also a precursor of hydrogen sulfide (H_2_S), which is produced by the transsulfuration pathway where cystathionine β-synthase (Cbs) catalyzes condensation of homocysteine and cysteine forming H_2_S, and hyperhomocysteinemia is associated with decreased H_2_S levels [17–19]. H_2_S acts as a gaseous mediator at physiological levels, but metabolic imbalance of homocysteine-H_2_S increases oxidative stress [20]. In diabetic retinopathy, retinal homocysteine metabolism is dysregulated, and while homocysteine levels are increased, H_2_S levels are decreased [21–23]. Our previous studies have shown that high homocysteine damages retinal mitochondria, and supplementation of a slow-releasing H_2_S donor, GYY4137, protects mitochondrial function and the development of retinopathy in streptozotocin-induced diabetic mice [24]. However, it is unclear if an imbalance in homocysteine-H_2_S also affects removal of the damaged mitochondria.

The aim of this study was to investigate if the presence of homocysteine in a hyperglycemic medium accelerates mitochondrial damage and impairs removal of the damaged mitochondria. The temporal relationship between hyperhomocysteinemia and mitochondrial quality control was determined in human retinal endothelial cells (HRECs) incubated in high glucose for 24 to 96 h. To examine the effect of homocysteine- H_2_S imbalance on mitochondrial homeostasis, the effect of a slow releasing H_2_S donor- GYY4137 [25] on the removal of the damaged mitochondria was investigated. In vitro results were confirmed in the retinal vasculature from a hyperhomocysteinemic mouse model with different durations of streptozotocin-induced diabetes.

## Methods

### Human retinal endothelial cells

HRECs (Cat. No. ACBRI 181, Cell Systems Corp., Kirkland, WA, USA) were cultured in Dulbecco’s modified Eagle Medium (DMEM) supplemented with fetal bovine serum, endothelial cell growth supplement, glutamax, insulin-transferrin-selenium and antibiotic/antimycotic. Cells (~ 80% confluent) from the 6th to 8th passage were incubated in 5 mM d-glucose (NG group), or in 20 mM d-glucose without (HG group) or with 100 µM l-homocysteine thiolactone hydrochloride (Cat. No. S784036; Sigma-Aldrich, St. Louis, MO, USA; Hcy group) for 24 to 96 h. Based on Hcy levels in diabetic patients ranging from 16 to over 100 µM (moderate to severe hyperhomocysteinemia), 100 µM homocysteine was used in our study; this concentration was shown to result in mitochondrial damage without having any effect on the cell phenotype [21, 24, 26]. To analyze the effect of H_2_S regulation, 150 µM GYY4137 (Cat. No. SML0100; Sigma-Aldrich) was added to a group of cells incubated with high glucose + homocysteine (GY group) as described previously [24, 27]. As an osmotic control, each experiment included cells incubated with 20 mM l-glucose (L-Gl group), instead of 20 mM d-glucose. Incubation medium was replaced every 48 h, and fresh homocysteine or GYY4137 was supplemented during each media change. Duplicate measurements were taken and each experiment was repeated with three to five different HREC preparations.

### Animals

Mice deficient in *Cbs*, a model of hyperhomocysteinemia, were generated by breeding *Cbs* heterozygous (*Cbs*^+*/−*^) mice (B6.129P2-Cbstm1Unc/J; Jackson Laboratories, Bar Harbor, ME, USA), and their genotyping was performed following the method reported by others [28, 29]. Since *Cbs*^*−/−*^ mice have a very short life span, studies were performed using C*bs*^+*/−*^ mice, and wildtype (*Cbs*^+*/*+^) mice were used as controls. Diabetes was induced in 8 to 10 weeks old *Cbs*^+*/−*^ and *Cbs*^+*/*+^ mice (male and female) by streptozotocin administration (55 mg/kg) for four consecutive days. Mice were maintained diabetic (blood glucose > 350 mg/dL) for eight weeks to six months. Age-matched nondiabetic mice served as the controls, and each group had 6–8 mice. The retina was isolated immediately and was used for measurement of biochemical/molecular parameters. For mice diabetic for over 16 weeks, the retina was isolated from one eye and the other eye was fixed in 10% buffered formalin for histopathology [30, 31]. Animal care and maintenance followed Wayne State University’s institutional guidelines for treating animals humanely, and Association for Research in Vision and Ophthalmology’s declaration for using animals in ophthalmic and vision research (protocol #21-03-3331). This study is reported in accordance with the Animal research: reporting of in vivo experiments (ARRIVE) guidelines.

### Retinal microvessels

Microvessels were prepared from the mouse retina following the hypotonic shock method. Briefly, the retina was placed in distilled water for an hour at 37 °C, and the vasculature was prepared as reported previously [27, 30].

### Homocysteine

Total homocysteine was measured in the retina (25 µg protein) using an ELISA kit (Cat. No. STA-670; Cell Bio Labs Inc., San Diego, CA, USA) using homocysteine-BSA as a standard, as reported previously [22].

### Activity of cystathione β synthase (Cbs)

Cbs activity was assessed utilizing 40 to 60 µg of retinal protein in accordance with the cystathionine-β-synthase activity assay kit (Cat. No. K998; Bio Vision, Milpitas, CA, USA) using cysteine and homocysteine as substrates [24]. To ascertain the specificity of Cbs activity, 7-amino-4-methylcoumarin was employed as a standard.

### Hydrogen sulfide

H_2_S was quantified using the methylene blue assay [22, 24]. Briefly, cell culture medium (250 µL) or retinal homogenate (50 µg protein) was incubated with 1% zinc acetate at room temperature for 20 min. This was followed by the addition of 20 mM N-dimethyl-p-phenylenediamine sulfate and 30 mM FeCl_3_, and the mixture was incubated at 37 °C for one hour in the dark. After precipitating the protein with 10% trichloroacetic acid, followed by centrifugation at 10,000x*g* for 5 min, the absorbance of the supernatant was measured at 670 nm. Sodium hydrosulfide (Cat. No. 161527; Sigma-Aldrich) was employed as a standard.

### Mitochondrial ROS

Mitochondrial ROS were quantified fluorometrically in retinal microvessels using MitoSox red (Cat. No. M36008; Thermo Fisher Scientific, Waltham, MA, USA). Cell homogenate (10 µg protein) was incubated in a microplate with 5 µM MitoSox red in the dark for 10 min at 37 °C, and the fluorescence was measured at excitation/emission wavelengths of 500 nm/580 nm. As a positive control, 5 mM d-glucose incubated cells were exposed to 2 μM antimycin A for one-hour before being analyzed.

### RNA extraction and mtDNA damage

Total RNA was extracted using TRIzol reagent as per manufacturer’s instructions (Cat. No. 15596026, Invitrogen, Waltham, MA, USA). Purity of the extracted RNA was assessed by quantifying the ratio of absorbance at 260 nm and 280 nm; a ratio of ~ 2.0 was considered pure. cDNA synthesis was processed after treating RNA with DNase I. Mitochondrial DNA (mtDNA) damage was evaluated in HRECs or retinal microvessels by quantifying gene transcripts of mtDNA-encoded NADH-ubiquinone oxidoreductase chain 6 (*ND6*) and cytochrome B (*CytB*) by SYBR-green based real-time polymerase chain reaction (PCR), as reported previously [32], using gene- and species-specific primers (Table 1). Relative expression of the gene was calculated by the Delta-Delta-Ct method using *β-actin* (HRECs) or *18S rRNA* (mouse) as the housekeeping gene [32].

**Table 1:** Primer sequence

Primer	Sequence (5’-3’)
*Human RNA*	
*CytB*	Fwd-TCACCAGACGCCTCAACCGC Rev-GCCTCGCCCGATGTGTAGGA
*ND6*	Fwd-GACCTCAACCCCTGACCCCCA Rev-GCGGTGTGGTCGGGTGTGTTAT
*B-actin*	Fwd-AGCCTCGCCTTTGCCGATCCG Rev-TCTCTTGCTCTGGGCCTCGTCG
*Human DNA*	
*CytB*	Fwd-TCACCAGACGCCTCAACCGC Rev-GCCTCGCCCGATGTGTAGGA
*B-actin*	Fwd-CTTTCCTGCCTGAGCTGACC Rev-CCTAGAAGCATTTGCGGTGG
*Mouse RNA*	
*Cbs*	Fwd-TTTGTCTCTAGCGCGTCACC Rev-TCCCGCTAGTATCGACCTCC
*CytB*	Fwd-ACCCGCCCCATCCAACATCTCAT Rev-TTGAGGCTCCGTTTGCGTGT
*ND6*	Fwd-TGGTTGGTTGTCTTGGGTTGGCA Rev-CCGCTACCCAATCCCTCCCT
*18S rRNA*	Fwd-GCCCTGTAATTGGAATGAGTCCACTT Rev-CTCCCCAAGATCCAACTACGAGCTTT
*Mouse DNA*	
*CytB*	Fwd-GCAACCTTGACCCGATTCTTCGC Rev-TGAACGATTGCTAGGGCCGCG
*18S rRNA*	Fwd-GCCCTGTAATTGGAATGAGTCCACTT Rev-CTCCCCAAGATCCAACTACGAGCTTT

### DNA isolation and mitochondrial copy numbers

Genomic DNA was isolated from HRECs or retinal microvessels using the DNeasy kit (Cat. No. 69504, Qiagen, Valencia, CA, USA) according to the manufacturer’s protocol. Mitochondrial copy numbers were quantified in the genomic DNA by SYBR-green based amplification of the mitochondrial marker, *CytB,* and nuclear marker (*β-actin* or *18S rRNA*), using DNA primers provided in Table 1 [32].

### Oxygen consumption rate (OCR)

A Seahorse XF analyzer (Agilent Technologies, Santa Clara, CA, USA) was employed to measure OCR. HRECs (~ 90% confluent) were trypsinized, and after washing the cell pellet with DMEM, it was suspended in the growth medium. The suspension (10 μL) was then loaded into a Neubauer chamber (Microyn Technologies, Baltimore, MD, USA) to count the cells, and was diluted to achieve a concentration of 50,000 cells/mL. Cell suspension (100 μL) was seeded in each well of the 96 well Seahorse culture plate. At the end of experimental treatments, HRECs were washed with 100 µL assay medium (Seahorse XF medium supplemented with 1 mM pyruvate, 2 mM glutamine and 10 mM glucose), and were then incubated at 37 °C for 30 to 45 min with 180 µL assay medium. OCR was measured using Seahorse XF Cell Mito Stress Test kit (Cat. No. 103015-100, Agilent Technologies) by injecting 1.5 µM oligomycin, 2.0 µM FCCP and 0.5 µM rotenone/antimycin A in ports A, B and C, respectively. The data was analyzed using the Wave software (Agilent Technologies), as reported recently [13].

### Mitophagosome formation

Mitophagosome formation was quantified by an Autophagy Detection kit (Cat. No. ab139484; Abcam, Boston, MA, USA). Following experimental incubations, cells were washed with the assay buffer (supplied by the manufacturer) containing 5% fetal serum and incubated with the green detection reagent in the dark for 30 min at room temperature. MitoTracker red and Hoechst were used to counterstain the mitochondria and nuclei, respectively. Cells were imaged under a Zeiss Apotome at 20 × objective, and arithmetic mean intensity (AMI) of green fluorescence was calculated using the Zeiss software module. Each experiment included rapamycin (1 µM) as an autophagy inducer control.

### Mitophagy flux

Mitophagy flux was assessed in live cells using the Mitophagy detection kit (Cat. No. MD01-10, Dojindo Molecular Technologies, Rockville, MD, USA). Briefly, cells were washed with DMEM and incubated with 100 nM Mtphagy dye for 30 min at 37 °C in dark. After washing the cells two times with FACS buffer (0.5% BSA in PBS w/v) to remove trypsin, scattering of Mtphagy Dye was quantified by flow cytometry under PerCP Cy5.5 channel at an excitation/emission wavelength of 486 nm/679 nm. Flow cytometry standard files were analyzed by FlowJo v.10.8.1 software (BD Biosciences, San Jose, USA), and relative Mtphagy scattering for each group was plotted, as recently reported [13]. As a positive control, 1 μM rapamycin was used.

Mitophagy flux in the mouse retina was quantified by flow cytometry [13]; briefly, tissue disaggregation was carried out by incubating small pieces of retina with 50 µL Accumax™ (Sigma-Aldrich, USA) for 10 min at 37 °C. The resultant suspension was filtered through a 40 µm cell strainer and incubated for 30 min at 37 °C with staining solution containing 50 nM MitoTracker deep red (MTDR, Cat No. M22426, Thermo Fisher Scientific). Cells were washed with FACS buffer (2X) and scanned under FL3 640 nm wavelength using BD Accuri C6 plus flow cytometer. Raw Flow Cytometry Standard files were analyzed employing FlowJo software, and relative MTDR scattering in each group was plotted, as reported recently [13].

### Retinal histopathology

Retina isolated from the formalin-fixed eyes was rinsed overnight in running water and incubated at 37 °C with 3% crude trypsin (Invitrogen-Gibco, Grand Island, NY, USA) containing 200 mM sodium fluoride for 45 to 70 min. After cleaning the retinal vasculature under a dissecting microscope, it was stained with periodic acid-Schiff-hematoxylin to count acellular capillaries [24, 31].

### Retinal vascular permeability

Permeability of retinal vasculature was determined by fluorescein angiography using a Micron IV retinal imaging microscope (Phoenix Research Laboratories, Pleasanton, CA, USA). After anesthetizing the animals with Ketamine-Xylazine (67 mg and 10 mg per kg, respectively; i.p.) and dilating pupils with 0.1% tropicamide ophthalmic solution and lubricating their corneas with Goniovisc (hypromellose 2.5%). The fundus photographs were taken by fundus camera for small animals. AK-FLUOR (0.5% solution, 0.01 mL/g BW) was then injected intraperitoneally, and the fundus was photographed using a barrier filter for fluorescein angiography, as reported previously [24, 31].

### Statistical analysis

GraphPad Prism was used to perform statistical analysis. Data are presented as mean ± standard deviation of three or more experiments. Inter-group comparisons were made using one-way ANOVA followed by post hoc Tukey test. *P* values were calculated using the Student’s t-test and *P* values less than 0.05 were considered statistically significant.

## Results

### Retinal endothelial cells in vitro

**Fig. 1:**
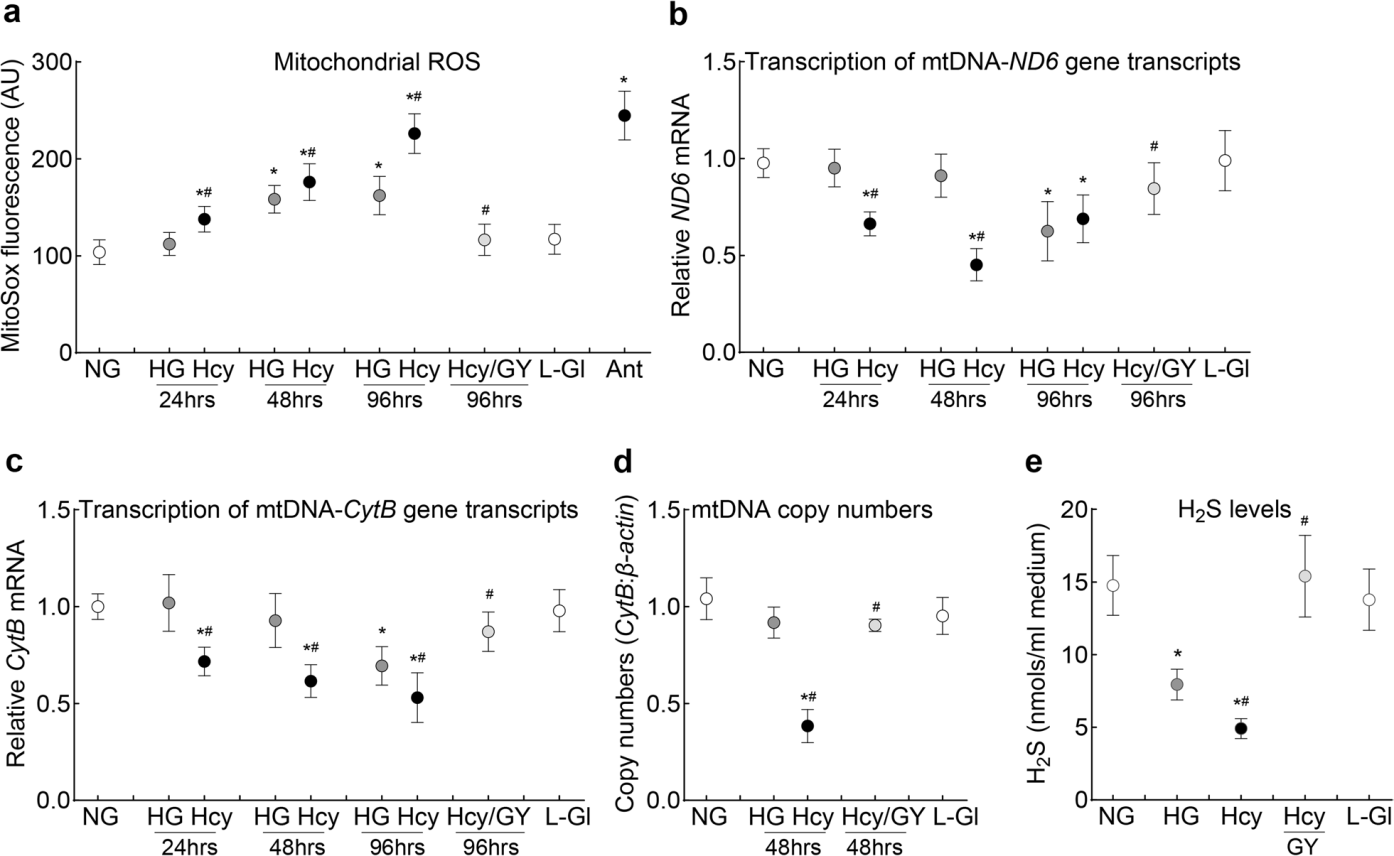
Temporal relationship between high glucose-homocysteine and mitochondrial damage in retinal endothelial cells. Human retinal endothelial cells were analyzed for (**a**) mitochondrial reactive oxygen species (ROS) using MitoSox red, gene transcripts of mtDNA-encoded (**b**) *ND6* and (**c**) *CytB* by quantitative reverse transcription polymerase chain reaction using *β-actin* as housekeeping gene, and (**d**) mitochondrial copy numbers in genomic DNA using *CytB* as a mtDNA marker and *β-actin* as a nuclear DNA marker. (**e**) H_2_S levels were quantified spectrophotometrically using N-dimethyl-p-phenylenediamine sulfate. Results are presented as mean ± SD from three different cell preparations, with each measurement made in triplicates. NG and HG, 5 mM and 20 mM d-glucose, respectively; Hcy and Hcy/GY, 20 mM d-glucose with homocysteine, without and with GYY4137, respectively; L-Gl, 20 mM l-glucose; Ant., 5 mM d-glucose with antimycin A. *P < 0.05 vs. NG; ^#^*P* < 0.05 vs. HG

Exposure of HRECs to high glucose for 24 h, as expected [33], had no effect on mitochondrial ROS levels, but within 48 h, ROS levels increased significantly, and remained elevated up to 96 h. However, supplementation of homocysteine in high glucose medium (Hcy group) accelerated and exacerbated mitochondrial ROS production; compared to cells in normal or high glucose, mitochondrial ROS levels were significantly elevated within 24 h (*P* < 0.05). Furthermore, compared to the HG group, ROS levels in the Hcy group were significantly higher at 96 h (*P* < 0.05, Fig. 1a). To investigate if ROS production is also accelerating mtDNA damage, transcripts of mtDNA-encoded *ND6* of Complex I and *CytB* of Complex III were quantified. Compared with cells cultured in normal glucose, gene transcripts of *ND6* and *CytB* were unchanged in cells exposed to high glucose for up to 48 h but these decreased significantly at 96 h (*P* < 0.05). However, homocysteine supplementation significantly decreased both *ND6* and *CytB* gene transcripts within 24 h (Figs. 1b, c). Consistent with ROS levels and mtDNA damage, copy numbers, as expected [32], also remained unchanged in cells exposed to high glucose for up to 48 h (Fig. 1d), but were significantly decreased in the Hcy group (*P* < 0.05). Extending the duration of incubation of HRECs with 5 mM d-glucose or 20 mM l-glucose from 24 to 96 h had no effect on any of the parameters, and the values represented in NG and L-Gl groups are the pooled values from 24 to 96 h of incubation. Furthermore, addition of GYY4137 in Hcy group produced consistent beneficial effects at 24 to 96 h of incubation; the data presented in the figures are from 96-h time point.

**Fig. 2:**
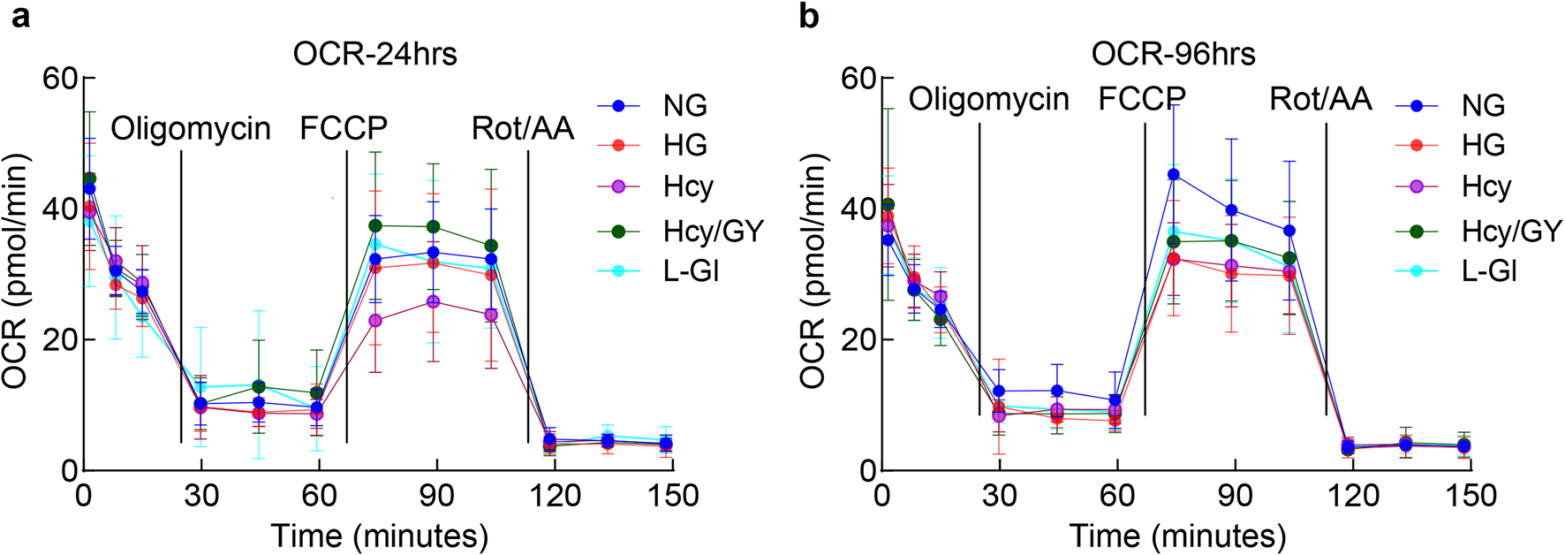
Mitochondrial stress and homocysteine. Oxygen consumption rate (OCR) was measured in human retinal endothelial cells incubated in high glucose-homocysteine for (**a**) 24 h and (**b**) 96 h using a Seahorse XF Cell Mito Stress Test kit. Measurements were repeated two times, with each measurement containing eight wells/group; the results in the graphs are presented as mean ± SD. NG, 5 mM d-glucose; HG and HG/Hcy, 20 mM d-glucose without and with homocysteine, respectively; Hcy/GY, 20 mM d-glucose with homocysteine and GYY4137; l-Gl, 20 mM l-glucose; FCCP, carbonyl cyanide p-trifluoromethoxyphenylhydrazone; Rot/AA, rotenone/antimycin A

Incubation of HRECs with high glucose, as reported previously [24], resulted in significant decrease in H_2_S, which was further worsened by the addition of homocysteine; values in the HG and Hcy groups were different from each other (*P* < 0.05), and were also significantly lower than those from the NG or L-Gl groups (Fig. 1e). Supplementation of the H_2_S donor GYY4137, in addition to ameliorating glucose or glucose + homocysteine induced decrease in H_2_S, attenuated ROS levels and mtDNA damage and prevented decrease in mtDNA copy numbers; values in the Hcy/GY group were not different from those obtained from the NG or L-Gl groups (*P* > 0.05). Effect of high homocysteine-high glucose on mitochondrial damage was further confirmed by measuring mitochondrial stress. High glucose exposure for 24 h had no significant effect on OCR, and the OCR patterns after addition of oligomycin, and stimulation by mitochondrial uncoupler carbonyl cyanide 4-[trifluoromethoxy] phenylhydrazone (FCCP), were similar in the NG and HG groups. However, the addition of homocysteine in a high glucose medium resulted in a significantly blunted effect, which was protected in the presence of GYY4137; OCR patterns in HG and Hcy/GY groups were not significantly different from each other (Fig. 2a). However, when high glucose insult was extended to 96 h, HG and HG/Hcy groups had similar blunted OCR patterns (Fig. 2b). Similar OCR patterns in HG and HG/Hcy groups after 96 h of incubation raises the possibility that the sustained high glucose insult, in the presence or absence of homocysteine, can result in a threshold OCR.

**Fig. 3:**
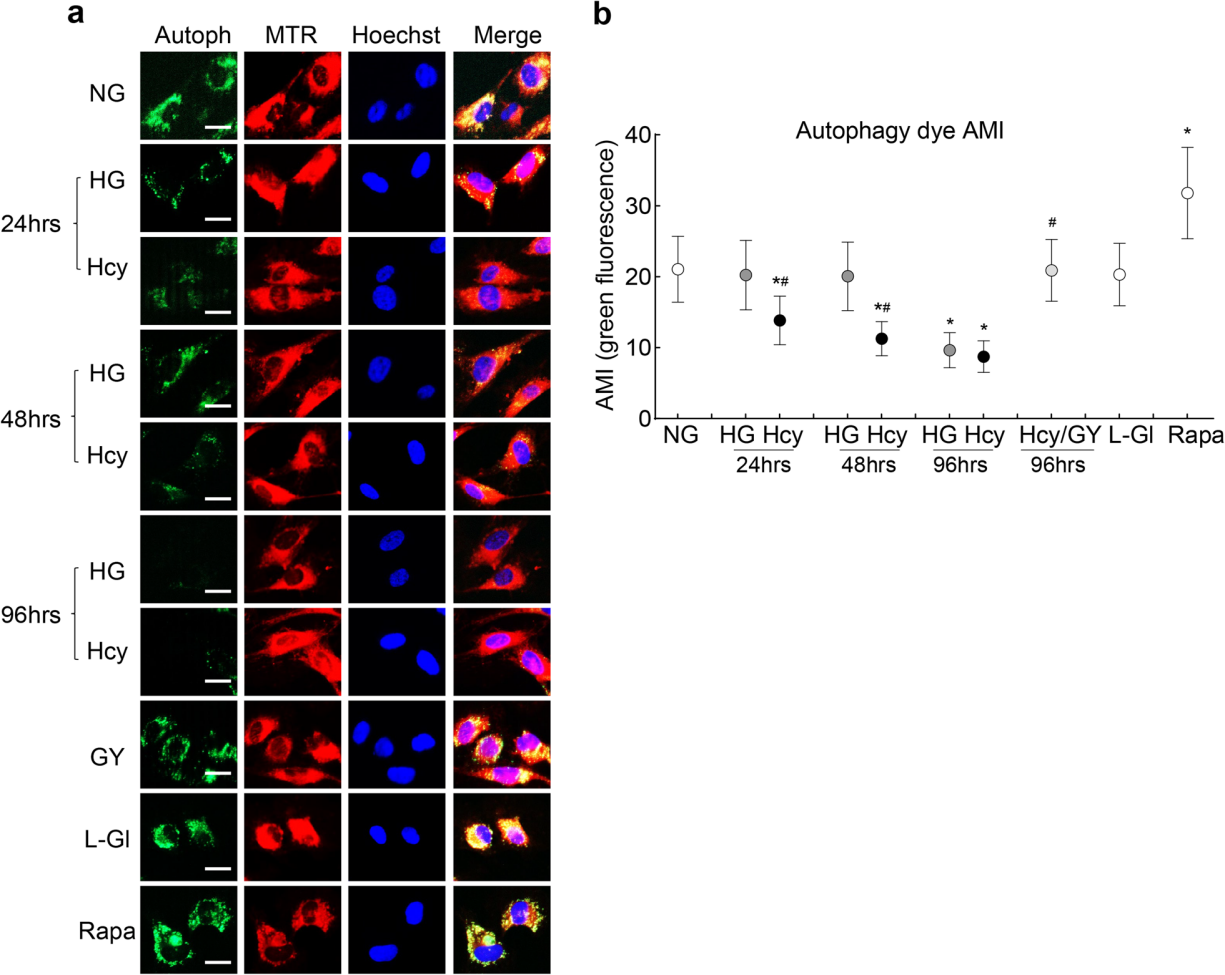
Glucose-homocysteine and mitophagy in retinal endothelial cells. Human retinal endothelial cells were analyzed for mitophagosome formation by (**a**) staining with an autophagy dye and visualizing with an Apotome using 20 × objective (scale bar = 20 μm). **b** Arithmetic mean intensity (AMI) of green fluorescence from three different cell preparations was plotted, and each measurement was made in triplicates. Green = mitophagosomes, blue = nucleus stained with Hoechst and red = mitochondria stained with MitoTracker red (MTR); NG and HG, 5 mM and 20 mM d-glucose, respectively; Hcy and Hcy/GY, 20 mM d-glucose with homocysteine, without and with GYY4137, respectively; l-Gl, 20 mM l-glucose; Rapa, 5 mM d-glucose with Rapamycin. *P < 0.05 vs. NG; ^#^*P* < 0.05 vs. HG

Damaged mitochondria continue to produce ROS and their removal is facilitated by the formation of mitophagosomes, which, after fusing with lysosomes, degenerates [34]. The retinal mitophagy process is impaired in diabetic retinopathy [13], therefore, the effect of homocysteine on the removal of damaged mitochondria was investigated. Compared to normal glucose, mitophagosome formation remained normal in cells exposed to high glucose for up to 48 h, but at 96 h, it was decreased by over 50%. Addition of homocysteine in a high glucose medium resulted in the decrease in mitophagosome formation within 24 h, and at 96 h, the values from the HG and HG/Hcy groups were not different from each other (*P* > 0.05) but were significantly less compared to the NG and L-Gl groups. The addition of GYY4137 ameliorated high glucose + homocysteine induced decrease in mitophagosomes (*P* < 0.05; Figs. 3a and b).

**Fig. 4:**
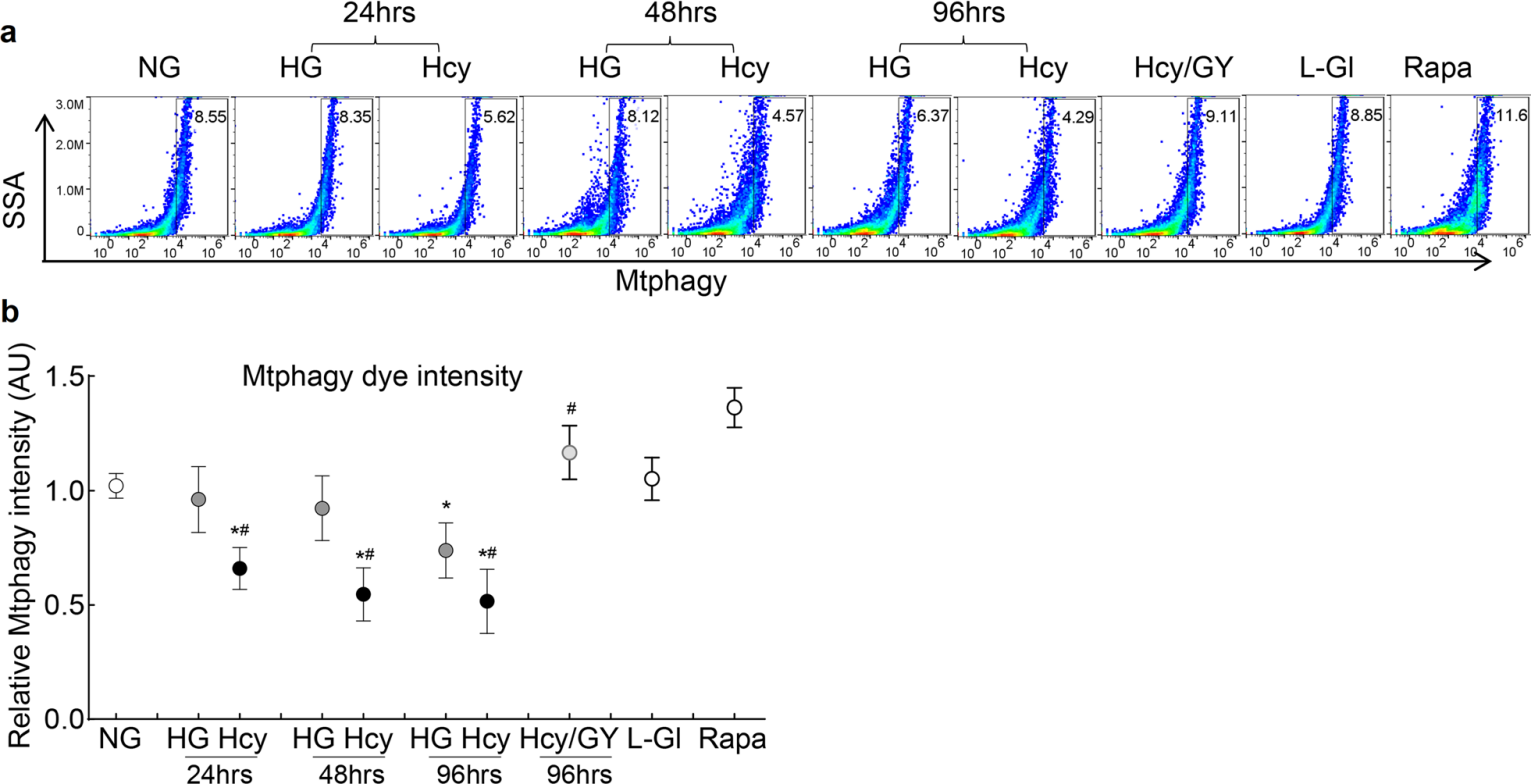
Mitophagy flux and homocysteine. Human retinal endothelial cells were (**a**) stained with Mtphagy dye and scanned under PerCP Cy5.5 channel in a BD Accuri C6 plus flow cytometer. **b** Plot showing relative fluorescence intensity of Mtphagy dye; results are presented as mean ± SD from three different cell preparations, with each measurement made in duplicates. NG, 5 mM d-glucose; HG and HG/Hcy, 20 mM d-glucose without and with homocysteine, respectively; Hcy/GY, 20 mM d-glucose with homocysteine and GYY4137; L-Gl, 20 mM l-glucose; Rapa, 5 mM d-glucose with rapamycin. *P < 0.05 vs. NG; ^#^*P* < 0.05 vs. HG

Consistent with mitophagosome formation, scattering of the Mtphagy dye, an indicator of mitophagy flux, was decreased in the Hcy group vs. NG group as early as 24 h of high glucose-homocysteine exposure, but this decrease was not observed till exposure to high glucose alone was extended to 96 h. GYY4137 supplementation in the Hcy group prevented decrease in mitophagy flux and the values obtained from cells in the Hcy/GY group, or in 20 mM l-glucose, were not different from each other, but were significantly different from those obtained from cells in 20 mM d-glucose, with or without homocysteine (Fig. 4a and b).

### In vivo, mouse model

**Fig. 5:**
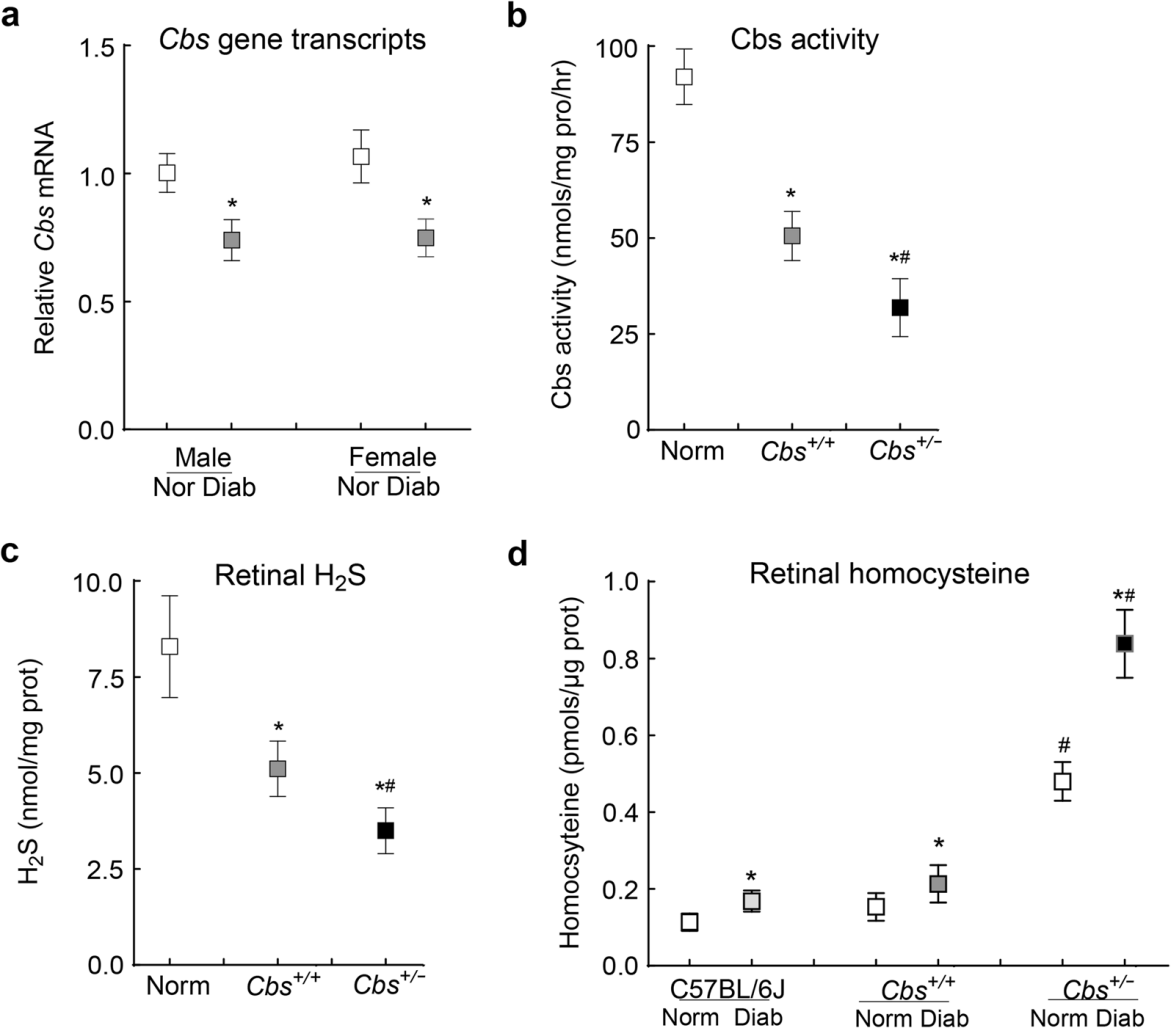
Effect of diabetes on Cbs activity and homocysteine metabolism. **a** *Cbs* gene transcripts were analyzed in the retina from C57BL/6 J mice diabetic for 24 weeks by quantitative reverse transcription polymerase chain reaction using 18S rRNA as the housekeeping gene. Retina from *Cbs*^+*/*+^ and *Cbs*^+*/−*^ mice diabetic for 24 weeks were analyzed for (**b**) Cbs activity using a fluorometric method, (**c**) H_2_S levels using N-dimethyl-p-phenylenediamine sulfate, and (**d**) total homocysteine levels via ELISA. Each measurement was performed in duplicate with 5–6 mice/group and results are presented as mean ± SD. Norm, normal *Cbs*^+*/*+^ mice; Diab, diabetes; *Cbs*^+*/*+^ and *Cbs*^+*/−*^, *Cbs* wildtype and heterozygous mice, respectively; *P < 0.05 vs. normal and ^#^*P* < 0.05 vs.* Cbs*^+*/*+^ diabetic mice

C57BL/6J male and female mice, diabetic for 24 weeks, had similar reduction (~ 25%) in retinal *Cbs* gene transcripts (Fig. 5a). In *Cbs*^+*/−*^ and *Cbs*^+*/*+^ diabetic mice, the severity of hyperglycemia was similar (blood glucose 350–500 mg/dl), and at 24 weeks of diabetes, gene transcripts of retinal *Cbs* were decreased by > 60% and ~ 40%, respectively, compared to their age-matched normal mice. Sex of the mice had no effect on the severity of hyperglycemia and retinal *Cbs* gene transcripts. In addition to *Cbs* gene transcripts, our previous studies have shown similar retinal histopathology and mitochondrial dysfunction in male and female mice [31], and the results presented here are pooled from both sexes.

Compared to normal mice*,* eight weeks of diabetes in *Cbs*^+*/*+^ mice resulted in ~ 40% reduction in retinal Cbs activity and H_2_S levels, and 25% to 30% increase in total homocysteine levels. However, in *Cbs*^+*/−*^ mice diabetic for eight weeks, retinal Cbs activity and H_2_S levels were downregulated by 60% to 75% and homocysteine levels were increased by ~ fourfold. The values were significantly different from those obtained from *Cbs*^+*/*+^ mice diabetic for eight weeks (*P* < 0.05; Fig. 5b–d).

**Fig. 6:**
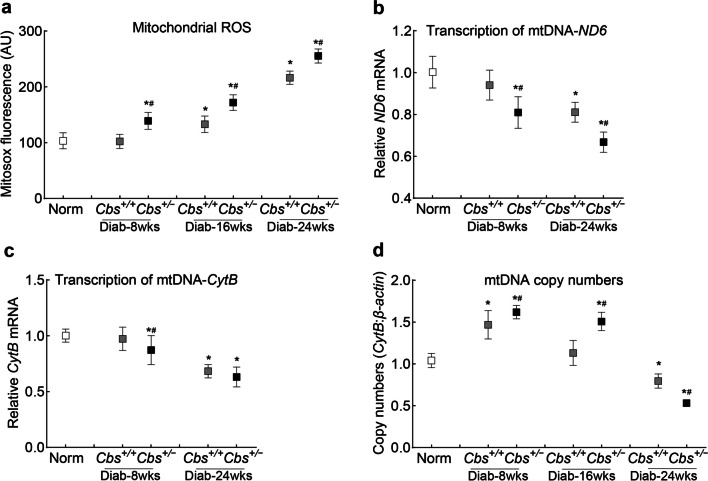
Temporal relationship between hyperglycemia-hyperhomocysteinemia and mitochondrial damage in mice. Retinal microvessels were analyzed for (**a**) mitochondrial reactive oxygen species (ROS) using MitoSox red, gene transcripts of (**b**) *ND6* and (**c**) *CytB* by quantitative reverse transcription polymerase chain reaction, and (**d**) mitochondrial copy numbers using mtDNA-encoded *CytB* and nuclear DNA-encoded *β-actin*. Each measurement was performed in triplicates with 4–6 mice/group, and the results are presented as mean ± SD. Norm, normal *Cbs*^+*/*+^ mice; *Diab*, diabetes; *Cbs*^+*/*+^*, Cbs* wildtype mice; *Cbs*^+*/−*^, *Cbs* heterozygous mice; *wks* weeks; *P < 0.05 vs. normal and ^#^*P* < 0.05 vs.* Cbs*^+*/*+^ diabetic mice

Mitochondrial ROS levels, compared to age-matched nondiabetic mice, remained unchanged in *Cbs*^+*/*+^ mice diabetic for eight weeks, but within 16 weeks, ROS levels were significantly elevated, and remained elevated at 24 weeks (*P* < 0.05). However, in *Cbs*^+*/−*^ mice, increases in ROS levels were seen as early as eight weeks of diabetes, and at 16 and 24 weeks, ROS levels were significantly higher compared to *Cbs*^+*/*+^ mice diabetic for the same duration. Consistent with mitochondrial ROS, at eight weeks of diabetes, mtDNA was not damaged in *Cbs*^+*/*+^ mice, but at 24 weeks, gene transcripts of mtDNA-encoded *ND6* and *CytB* were significantly decreased. However, in *Cbs*^+*/−*^ mice, *ND6* and *CytB* gene transcripts were significantly decreased within eight weeks of diabetes (Fig. 6a–c). As expected, due to a compensatory mechanism [35], compared to normal mice, mitochondrial copy numbers were significantly higher within eight weeks of diabetes in *Cbs*^+*/−*^ and *Cbs*^+*/*+^ mice (*P* < 0.05). Within 16 weeks of diabetes, although *Cbs*^+*/*+^ mice had similar copy numbers as normal mice, they remained significantly higher in *Cbs*^+*/−*^ mice (*P* < 0.05). However, within 24 weeks of diabetes mtDNA copy numbers were decreased significantly in both *Cbs*^+*/−*^ and *Cbs*^+*/*+^ mice, and the values in the *Cbs*^+*/−*^ group remained lower than those in the *Cbs*^+*/*+^ group (*P* < 0.05; Fig. 6d).

**Fig. 7:**
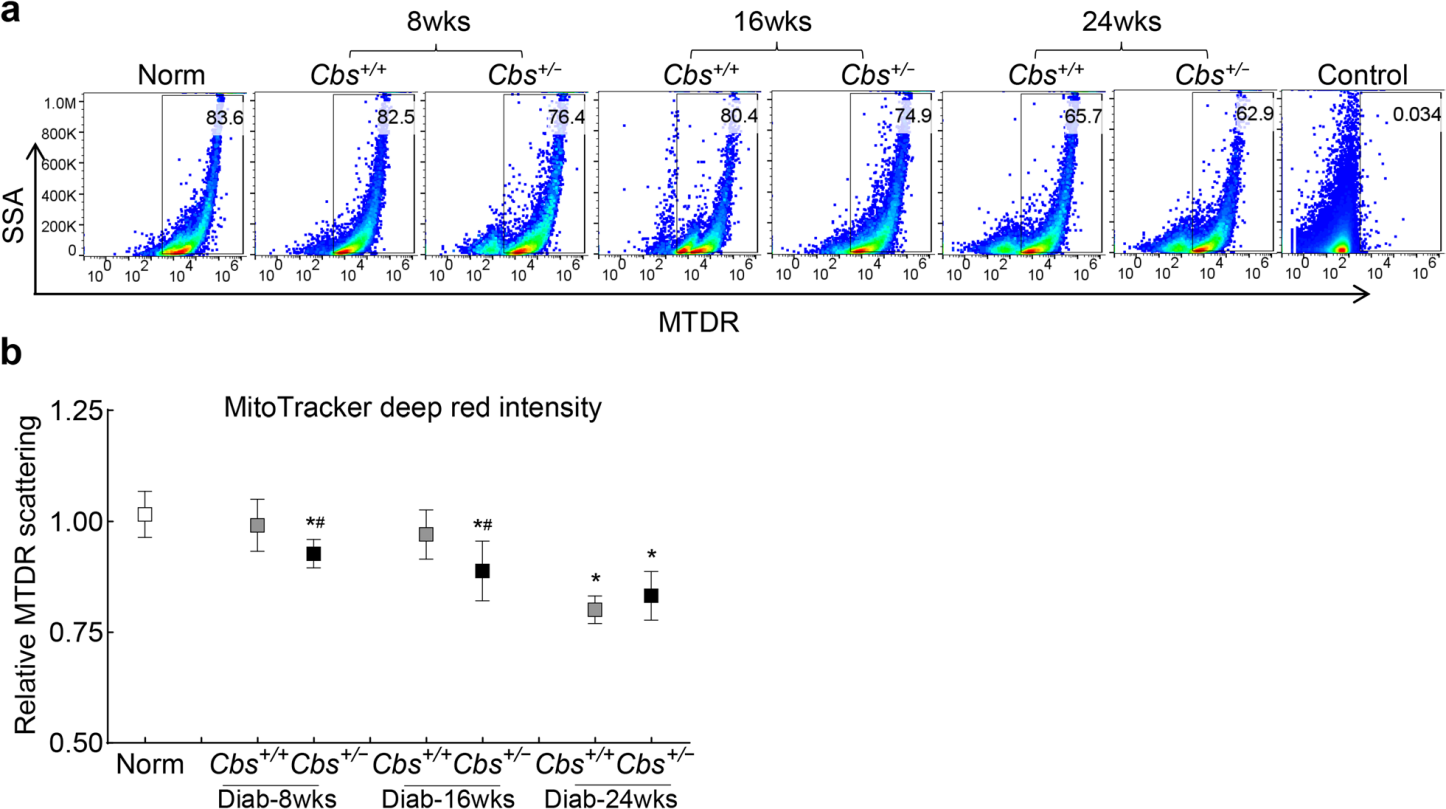
Mitophagy and hyperhomocysteinemia-hyperglycemia. **a** Mitophagy flux was analyzed by flow cytometry by staining retinal cells with MitoTracker deep red (MTDR). Each measurement was done in duplicate using 3–4 mice/group; **b** Relative fluorescence intensity of MTDR is presented as mean ± SD. Norm, normal *Cbs*^+*/*+^ mice; *Diab*, diabetes; *Cbs*^+*/*+^ and *Cbs*^+*/−*^ , Cbs wildtype and heterozygous mice, respectively; *wks* weeks; *P < 0.05 vs. normal and ^#^*P* < 0.05 vs.* Cbs*^+*/*+^ diabetic mice

In accordance with our in vitro results, compared with normal mice, retinal mitophagy flux in *Cbs*^+*/−*^ mice was decreased within eight weeks of diabetes vs. 24 weeks of diabetes in *Cbs*^+*/*+^ mice. At 16 weeks of diabetes, mitophagy flux in *Cbs*^+*/*+^ mice was similar to that in normal mice but was significantly lower in *Cbs*^+*/−*^ mice (*P* < 0.05), suggesting a poor and delayed removal of the damaged mitochondria. However, at 24 weeks of diabetes, mitophagy flux was similar in both *Cbs*^+*/*+^ and *Cbs*^+*/−*^ mice but was significantly lower than that in normal mice (Fig. 7a, b).

**Fig. 8:**
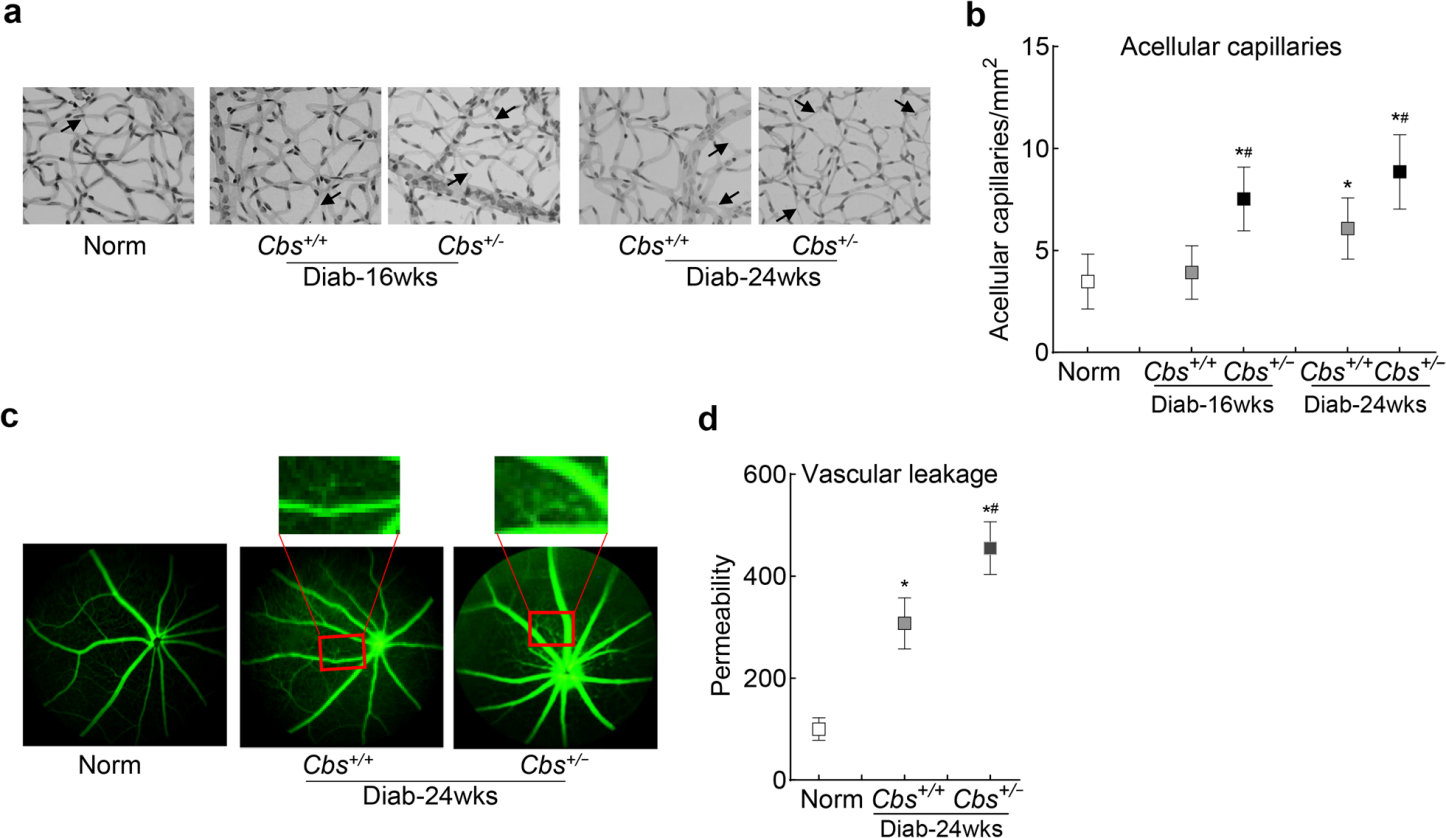
Retinal vascular damage in hyperhomocysteinemia-hyperglycemia. **a** Trypsin-digested retinal microvasculature was stained with periodic acid Schiff-hematoxylin; arrows indicate acellular capillaries and, (**b**) histogram representing the acellular capillary count in the entire retinal vasculature. **c** Fluorescein angiography was performed using a Micron IV retinal imaging microscope, and (**d**) the plot represents retinal vascular permeability, presented as mean ± SD from 4–6 mice/group*.* Norm, normal *Cbs*^+*/*+^ mice; *Diab* diabetes; *Cbs*^+*/*+^*, Cbs* wildtype mice; *Cbs*^+*/−*^, *Cbs* heterozygous mice; *wks*, weeks; *P < 0.05 vs. normal and ^#^*P* < 0.05 vs.* Cbs*^+*/*+^ diabetic mice

The number of acellular capillaries in the retinal vasculature of *Cbs*^+*/*+^ mice diabetic for about 16 weeks was similar to that observed in nondiabetic mice, but the numbers increased significantly when the duration of diabetes was extended to 24 weeks. In *Cbs*^+*/−*^ mice, significant increase in acellular capillaries was observed within 16 weeks of diabetes, and after 24 weeks of diabetes, their numbers were over 30% higher, compared to *Cbs*^+*/*+^ mice with similar duration of diabetes (Fig. 8 a and 8b). Consistent with retinal histopathology, vascular health, as evaluated by fluorescein angiography, showed vascular leakage in *Cbs*^+*/*+^ mice diabetic for 24 weeks, but the leakage was significantly higher in *Cbs*^+*/−*^ diabetic mice (*P* < 0.05; Fig. 8 c and d).

## Discussion

Diabetic retinopathy has a multi-factorial etiology, and impairment in mitochondrial homeostasis is considered to play a major role in its development [2, 4]. Mitochondrial homeostasis involves coordination of mitochondrial biogenesis, dynamics and mitophagy [7, 8], and all of these processes are compromised/damaged in diabetic retinopathy, resulting in the accumulation of damaged mitochondria and increased superoxide production [9]. Damaged mitochondria accelerate the apoptosis of retinal capillary cells, leading to the formation of degenerative capillaries, an early hallmark of diabetic retinopathy [36, 37]. Although hyperglycemia is the main instigator of the development of diabetic retinopathy, many systemic factors also contribute in its pathogenesis [38]. Diabetic patients routinely present high circulating levels of homocysteine, a sulfur containing amino acid, and elevated homocysteine is now an emerging risk factor for diabetic retinopathy and cardiovascular disease [39]. Homocysteine dysmetabolism is closely associated with H_2_S, a gaseous signaling molecule, and impairments in transsulfuration machinery have been shown to result in decreased H_2_S levels [17–19]. Our previous studies demonstrated that human donors with documented diabetic retinopathy have three-fold increase in retinal homocysteine and 50% decrease in H_2_S levels, compared to their age- and sex- matched nondiabetic donors [22], and high homocysteine damages mitochondria including its fusion-fission process [40]. Here, we provide intriguing data showing that the presence of homocysteine in hyperglycemic medium accelerates production of mitochondrial ROS and mtDNA damage and slows down mtDNA biogenesis and removal of damaged mitochondria. Amelioration of decrease in H_2_S by a slow releasing H_2_S donor protects the mitochondria from hyperglycemia-induced increase in mtDNA damage and decrease in copy numbers and mitophagy. This study clearly suggests that retinal mitochondria in a hyperhomocysteinemic-hyperglycemic environment face a double whammy—while mitochondrial damage is accelerated, the removal of the damaged mitochondria also slows down, and maintenance of a balance between homocysteine and H_2_S protects mitochondria from high homocysteine-hyperglycemic insult.

Increase in mitochondrial ROS damages mtDNA and impairs the transcription of mtDNA-encoded genes, resulting in a compromised electron transport chain system [2, 41]. Here, we show that increase in mitochondrial ROS and mtDNA damage and decrease in mitochondrial respiration in retinal endothelial cells is accelerated and exacerbated when homocysteine is supplemented in a high-glucose milieu. These results are supported by reports showing high homocysteine-induced damage of retinal endothelial cell barrier via oxidative stress [21], and electron transport chain damage in Parkinson’s disease and in ischemic brain injury [42, 43]. Furthermore, ROS also damages mtDNA, and thus self-propagate a vicious cycle of free radicals production [2, 4], where ROS can induce cellular and molecular oxidative injury in a hyperhomocysteinemic environment [44]. Taken together, these results strengthen the importance of maintaining low homocysteine levels in a diabetic patient.

As mentioned above, mitochondrial homeostasis involves a cascade of independent, but closely related processes, and a complex balance between mitochondrial dynamics, biogenesis and removal of the damaged mitochondria maintains mitochondrial quality control [7]. Mitochondrial biogenesis is not only associated with cell division, it also responds to oxidative stimuli and increase in the energy requirements of the cells [45]. Here, we demonstrate that hyperglycemia, in a high homocysteine environment, in addition to accelerating mitochondrial damage, also slows down mitochondrial biogenesis (copy numbers). Mitochondrial biogenesis and mitochondrial dynamics have been shown to be reciprocally coupled [45]. Our previous work demonstrated that homocysteine presence in a hyperglycemic medium also impairs mitochondrial dynamics, resulting in the accumulation of fragmented mitochondria [40], supporting our finding of impaired biogenesis in this study.

Accumulation of poorly functional/damaged mitochondria increases ROS and decreases adenosine triphosphate production [8, 46, 47], making their removal critical for cell survival. Damaged mitochondria are engulfed by mitophagosomes, and mitophagosomes fuse with lysosomes to degrade the damaged mitochondria [9–11], and mitophagy process is impaired in diabetic retinopathy [13]. Decrease in mitophagy is implicated in many chronic diseases including diabetic corneal endothelial dysfunction and diabetic retinopathy [48–50]. Here, we show that decrease in the formation of mitophagosomes and mitophagic flux is experienced at a shorter duration of high glucose-homocysteine insult (24 h), compared to high glucose alone (96 h), suggesting that the poor turnover/removal of the damaged mitochondria, which retinal mitochondria experience in a hyperglycemic milieu, is further worsened in a high homocysteine environment. As increased accumulation of the damaged mitochondria results in apoptosis, in support of poor removal of the damaged mitochondria in hyperhomocysteinemia, our previous work has shown that supplementation of homocysteine in HRECs further increases glucose-induced ROS levels and apoptosis [26], further supporting worsening of mitophagy by homocysteine supplementation.

Homocysteine catabolism is associated with the formation of H_2_S, a bioactive compound controlling various physiological processes [17] and H_2_S is considered an important signaling molecule (third gaseous) with roles in a wide range of physiological and pathological conditions [51, 52]. It protects brain endothelial cells from methionine-induced oxidative stress and decline in its levels contributes to endothelial dysfunction in cerebral ischemia/reperfusion [53, 54]. Both human and animal models have demonstrated the role of H_2_S in vascular inflammation and cardiomyopathy [55]. In diabetes while plasma homocysteine levels are elevated, H_2_S levels are decreased [56–58], and administration of H_2_S improves wound healing via restoration of their endothelial progenitor cell function [59]. We have shown that prevention of decrease in H_2_S levels by a slow releasing H_2_S donor ameliorates diabetes-induced mitochondrial damage, and also inhibits the development of histopathology, characteristic of diabetic retinopathy and vascular leakage [24]. Here, we show that GYY4137 also prevents decrease in mitochondrial copy numbers and improves removal of the damaged mitochondria, further strengthening the importance of homocysteine-H_2_S balance in mitochondrial homeostasis-diabetic retinopathy. We recognize that GYY4137 is a hypotensive agent with vasorelaxant properties [60], our previous study has shown that intraperitoneal administration of GYY4137 for six months in diabetic mice prevents the development of histopathology characteristic of diabetic retinopathy without any deleterious effects on their glucose levels and their overall well-being including loss in body weight [24]. Furthermore, consistent with our results presented here, H_2_S attenuates homocysteine-induced mitochondrial ROS and improves respiration in osteoblastic cells [61], and in diabetic nephropathy, increases sirtuin 1 and decreases oxidative stress [62].

Our data from retinal endothelial cells are strongly supported by results from diabetic mice with hyperhomocysteinemia; mice genetically manipulated to experience elevated homocysteine levels in the retina (*Cbs*^+*/−*^ mice), have accelerated and exacerbated mitochondrial damage with increase in ROS, and mtDNA damage at shorter duration of diabetes, compared to hyperglycemic mice without hyperhomocysteinemia (*Cbs*^+*/*+^ mice). In support, high homocysteine levels are associated with damaged blood-retinal barrier and ischemia [63]. Moreover, removal of damaged mitochondria by mitophagy is also slow in *Cbs*^+*/−*^mice resulting in the accumulation of the damaged mitochondria. As mentioned above, accelerated retinal capillary cell apoptosis is intimately associated with mitochondrial dysfunction, which precedes the development of acellular capillaries [5, 6]. Furthermore, we show that high homocysteine, in a hyperglycemic environment, also accelerates and exacerbates retinal vascular damage, characteristic of retinopathy; in *Cbs*^+*/−*^ diabetic microvascular leakage and histopathology in the retina is seen at a shorter duration of diabetes, compared to the hyperglycemic model. This further strengthens the importance of regulating homocysteine levels for a diabetic patient during the early stages of diabetes to prevent further progression of retinopathy and preserve vision.

We acknowledge that *Cbs*^+*/−*^ mice used in our study were not retina-specific, and the effect of *Cbs* repression in other tissues on retinal damage cannot be ruled out; we plan to establish a retina-specific *Cbs* repression mouse model for our future studies. The present study also did not include cells in normal glucose + homocysteine or nondiabetic *Cbs*^+*/−*^ mice to investigate the effect of hyperhomocysteinemia alone; our previous study has shown that homocysteine in normal glucose condition, or in nondiabetic hyperhomocysteinemic mouse model, does not cause significant mitochondrial damage [26]. Furthermore, although the current study reports data from experimental models (in vitro and in vivo), our previous work showing effect of hyperhomocysteinemia on retinal mitochondrial stability in the donors with documented diabetic retinopathy [22], raises the possibility of similar impairments in the mitophagy process in patients with diabetic retinopathy.

## Conclusion

**Fig. 9:**
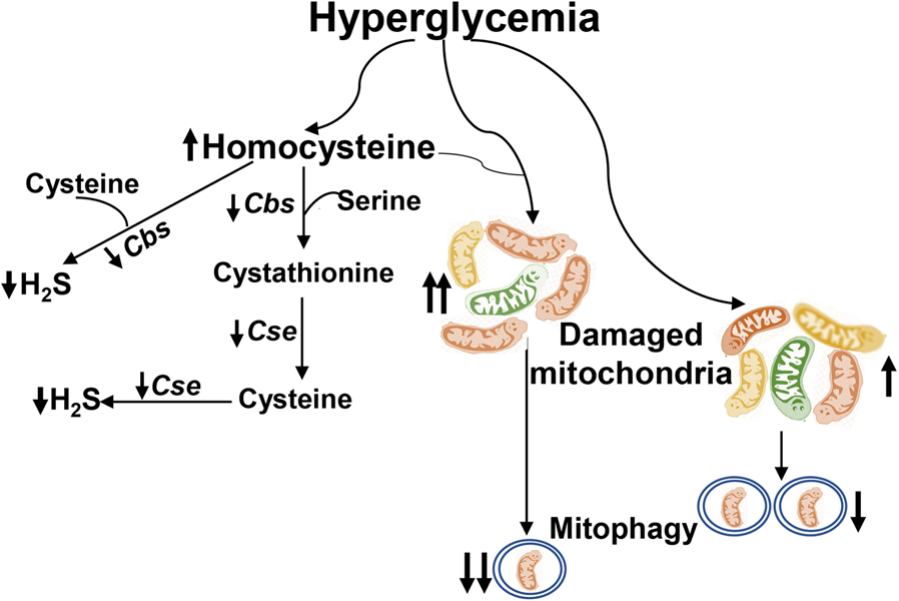
Working model. Diabetes elevates homocysteine and reduces H_2_S levels by impairing the transsulfuration pathway (inhibition of enzyme activities of Cbs and cystathionine γ-lyase, Cse). Presence of high homocysteine in hyperglycemic environment exacerbates and accelerates mitochondrial damage, and also decreases and slows down the removal of the damaged mitochondria (mitophagy), resulting in the exacerbation and acceleration of the development of diabetic retinopathy

Our results demonstrate that hyperhomocysteinemia accelerates mitochondrial damage, and also worsens/slows down their removal, further worsening their quality control and augmenting the development of diabetic retinopathy (Fig. 9). Our previous work has shown that high homocysteine in a diabetic environment affects mitochondrial homeostasis by damaging its DNA, biogenesis and dynamics by epigenetically modifying the genes associated with these processes [22–24, 26, 40], further confirming the damaging effects of homocysteine on diabetic retinopathy. Diabetes also impairs the balance between homocysteine and H_2_S, reducing H_2_S content [21, 22], and supplementation with a H_2_S donor prevents accumulation of the dysfunctional mitochondria, preventing/retarding the development of diabetic retinopathy. Thus, our results further strengthen the importance of maintaining proper balance between homocysteine and H_2_S for diabetic patients to protect them from this debilitating sight threatening complication, which affects over 80% of patients after 20 years of diabetes.

## Data Availability

RAK has full access to all the data in the study, and data are available on reasonable request.
